# European Society of Pediatric Radiology survey of perioperative imaging in pediatric liver transplantation: (2) intraoperative imaging

**DOI:** 10.1007/s00247-023-05840-1

**Published:** 2024-01-13

**Authors:** Jochen Herrmann, Philippe Petit, Stéphanie Franchi-Abella, Martijn V. Verhagen, Simon P. McGuirk, Elena Dammann, Reinoud P. H. Bokkers, Philippe R. M. Clapuyt, Annamaria Deganello, Francesco Tandoi, Jean de Ville de Goyet, Hanna Hebelka, Charlotte de Lange, Cecile Lozach, Paolo Marra, Darius Mirza, Piotr Kaliciński, Janina M. Patsch, Giulia Perucca, Ilias Tsiflikas, Diane M. Renz, Bernd Schweiger, Marco Spada, Seema Toso, Loïc Viremouneix, Helen Woodley, Lutz Fischer, Lil-Sofie Ording-Müller, Florian Brinkert

**Affiliations:** 1 Section of Pediatric Radiology, Department of Diagnostic and Interventional Radiology and Nuclear Medicine, Universitätsklinikum Hamburg-Eppendorf, Martinistrasse 52, 20246 Hamburg, Germany; 2 Department of Pediatric Radiology, Hôpital de La Timone: Hopital de La Timone, Marseille, France; 3 Department of Pediatric Radiology, Hôpital Bicêtre, Paris, France; 4 Department of Radiology, University Medical Centre Groningen: Universitair Medisch Centrum Groningen, Groningen, Netherlands; 5 Department of Radiology, Birmingham Children’s Hospital, Birmingham, UK; 6 Department of Radiology, Cliniques Universitaires Saint-Luc, Brussels, Belgium; 7 Department of Radiology, King’s College Hospital, London, UK; 8 Department of Hepatobiliary and Transplant Surgery, Azienda Ospedaliero-Universitaria Città Della Salute E Della Scienza Di Torino, Turin, Italy; 9 Department of Pediatrics and Pediatric Transplantation, ISMETT-UPMC, Palermo, Italy; 10 Department of Radiology, The Institute of Clinical Sciences, Gothenburg, Sweden; 11 Department of Pediatric Radiology, Queen Silvia Children’s Hospital: Sahlgrenska Universitetssjukhuset Drottning Silvias Barn- Och Ungdomssjukhus, Gothenburg, Sweden; 12 Department of Radiology, Hôpital Universitaire Necker-Enfants-Malades, Paris, France; 13 Department of Radiology, Azienda Ospedaliera Ospedali Riuniti Di Bergamo: Aziende Socio Sanitarie Territoriale Papa Giovanni XXIII, University of Milano-Bicocca, Bergamo, Italy; 14 Department of Hepatobiliary and Transplant Surgery, Birmingham Children’s Hospital, Birmingham, UK; 15 Department of Pediatric Surgery and Organ Transplantation, The Children’s Memorial Health Institute, Warsaw, Poland; 16 Department of Radiology, Medical University of Vienna, Vienna, Austria; 17 Department of Radiology, Great Ormond Street Hospital for Children, London, UK; 18 Department of Pediatric Radiology, Regina Margherita Children’s Hospital, Turin, Italy; 19 Department of Radiology, University Clinic of Tübingen, Tübingen, Germany; 20 Department of Pediatric Radiology, Hannover Medical School: Medizinische Hochschule Hannover, Hannover, Germany; 21 Department of Radiology, Institute of Diagnostic and Interventional Radiology and Neuroradiology, University Clinic of Essen, Essen, Germany; 22 Division of Hepatobiliopancreatic Surgery, Liver and Kidney Transplantation, Ospedale Pediatrico Bambino Gesu, Rome, Italy; 23 Department of Pediatric Radiology, Geneva University Hospitals: Hopitaux Universitaires Geneve, Geneva, Switzerland; 24 Department of Radiology, Hôpital Femme Mère Enfant - Hospices Civils de Lyon, Bron, France; 25 Department of Pediatric Radiology, Leeds Children’s Hospital, Leeds, UK; 26 Department of Visceral Transplant Surgery, Universitätsklinikum Hamburg-Eppendorf, Hamburg, Germany; 27 Department of Pediatric Radiology, Rikshospitalet University Hospital: Oslo Universitetssykehus Rikshospitalet, Oslo, Norway; 28 Department of Pediatric Gastroenterology and Hepatology, Universitätsklinikum Hamburg-Eppendorf, Hamburg, Germany

**Keywords:** Child, Computed tomography, Liver transplantation, Magnetic resonance imaging, Ultrasonography

## Abstract

**Background:**

Liver transplantation is the state-of-the-art curative treatment for end-stage liver disease. Imaging is a key element in the detection of intraoperative and postoperative complications. So far, only limited data regarding the best radiological approach to monitor children during liver transplantation is available.

**Objective:**

To harmonize the imaging of pediatric liver transplantation, the European Society of Pediatric Radiology Abdominal Taskforce initiated a survey addressing the current status of imaging including the pre-, intra- and postoperative phase. This paper reports the responses related to intraoperative imaging.

**Materials and methods:**

An online survey, initiated in 2021, asked European centers performing pediatric liver transplantation 48 questions about their imaging approach. In total, 26 centers were contacted, and 22 institutions from 11 countries returned the survey.

**Results:**

Intraoperative ultrasound (US) is used by all sites to assess the quality of the vascular anastomosis in order to ensure optimal perfusion of the liver transplant. Vessel depiction is commonly achieved using color Doppler (95.3%). Additional US-based techniques are employed by fewer centers (power angio mode, 28.6%; B-flow, 19%; contrast-enhanced US, 14.3%). Most centers prefer a collaborative approach, with surgeons responsible for probe handling, while radiologists operate the US machine (47.6%). Less commonly, the intraoperative US is performed by the surgeon alone (28.6%) or by the radiologist alone (23.8%). Timing of US, imaging frequency, and documentation practices vary among centers.

**Conclusion:**

Intraoperative US is consistently utilized across all sites during pediatric liver transplantation. However, considerable variations were observed in terms of the US setup, technique preferences, timing of controls, and documentation practices. These differences provide valuable insights for future optimization and harmonization studies.

**Supplementary information:**

Supplementary material is available at 10.1007/s00247-023-05840-1.

## Introduction

Liver transplantation is the state-of-the-art therapy for end-stage liver disease in children. Advances in organ procurement, surgical techniques, and immunosuppression have led to excellent short- and long-term results with a 5-year patient survival rate exceeding 85% [1–3]. Radiologic imaging methods are a key element to most transplantation programs as they have been shown to assist surgical planning, to guide intraoperative surgical technique, and can be effectively applied to detect postoperative complications [4–8].

The main objective for intraoperative imaging is to evaluate the inflow and outflow from the graft to ensure the hepatic vascular supply is optimized. In children, vascular complications are more frequent than in adults because of smaller vessels and caliber mismatch, often requiring vascular reconstruction [9–11]. Ultrasound (US) can easily be applied intraoperatively to assess hepatic in-flow and out-flow qualitatively and quantitatively at multiple time points during the transplantation. Application of a variety of different Doppler- and non-Doppler-based vascular imaging techniques including contrast-enhanced US (CE-US) have been reported [12–17].

So far, only a limited body of literature exists regarding the optimal setup for intraoperative US imaging during pediatric liver transplantation [18]. Quantitative data largely relies on the chosen methodology, necessitating strict standardization for meaningful interpretation. In an attempt to harmonize imaging among the European centers for pediatric liver transplantation, the European Society of Pediatric Radiology (ESPR) Abdominal Taskforce initiated an online survey addressing the practices of pre-, intra-, and postoperative imaging [19, 20]. This paper reports the responses on the intraoperative imaging section of the survey in order to find a common basis for later consensus recommendations and for multicenter studies.

## Material and methods

### The survey

This online survey by the ESPR Abdominal Taskforce contacted European centers for pediatric liver transplantation and asked about their current protocols regarding diagnostic imaging procedures. The survey followed a multidisciplinary approach, and the questions were directed towards all pediatric disciplines involved (e.g., radiology, transplantation surgery, gastroenterology, intensive care). A representative from each center was asked to gather the information from all sub-disciplines and to fill out the online survey using Google Forms. The survey was initiated in the year 2021 and the participating centers were asked to specify their liver transplantation numbers and choice of modalities for the period 2018–2020. A total of 48 questions were organized within six sections: demographics (seven questions), pre-transplant evaluation (eight questions), intraoperative imaging (eight questions), postoperative imaging (15 questions), liver elastography (six questions), and outlook (four questions). All survey questions can be found in Supplementary Material 1. For the questions on intraoperative imaging, see Table 1***.*** Further information on the participating centers and European site demographics can be found in the paper reporting the responses for preoperative imaging [19].

**Table 1:** Survey questions regarding intraoperative ultrasound

**1. Do you use intraoperative ultrasound (US) to control your vascular anastomoses?**	
	*no / yes, in every patient / yes, in some patients: please specify (free text)*
2. If yes: Who performs the intraoperative US?*	
	*transplant surgery alone / joint set-up: transplant surgery with radiology, with gastroenterology, with anaesthesiology / radiology alone*
**3. Do you save and store intraoperative ultrasound images to document the results?**	
	*no / yes, in every patient / yes, when performed by radiologist / other: (free text)*
**4. Do you save and store cine images (2-D/3-D volumes) to document the results?**	
	*no / yes, in every patient / yes, when performed by radiologist / other: (free text)*
**5. Do you perform intraoperative US at defined time points during the operation?**	
	*no / yes, after hepatic artery anastomosis / yes, after biliary anastomosis / yes, before and after abdominal closure / other: (free text)*
**6. Which US based vascular imaging technique do you use intra-operatively?**	
	*Color Doppler / Power angio mode / B-flow / Microvascular flow / other: (free text)*
**7. Do you additionally use US contrast intra-operatively?**	
	*no / yes, in every patient / only in case of unclear situations*
**8. Do you use other modalities or devices to monitor your vascular anastomoses?**	
	*no / yes: (please specify in free text)*

The question “Who performs the intraoperative US” was slightly adapted from the initial survey (see Supplementary Material 1) after some centers indicated “radiology alone” as their preferred set-up. This choice was included in the response option, and presented to all centers

*D* dimensional

## Results

### Setup and technique

Intraoperative US is the main method to assess the patency of vascular anastomosis and was used at all sites (21/21 sites, 100%). The most applied technique for vascular imaging is color Doppler US (20/21 sites, 95.3%) followed by power angio mode (6/21 sites, 28.6%), and B-flow (4/21 sites, 19%). Power angio mode and B-flow technology are usually applied in addition to color Doppler. For selected cases with complicated vascular anastomosis, contrast-enhanced (CE)-US is used at 3/21 sites (14.3%). Four sites (9.1%) apply additional intraoperative tools including perivascular pressure measurement, transit time flow measurement, liver parenchyma lactate monitoring, and intraoperative angiography in selected cases.

**Fig. 1:**
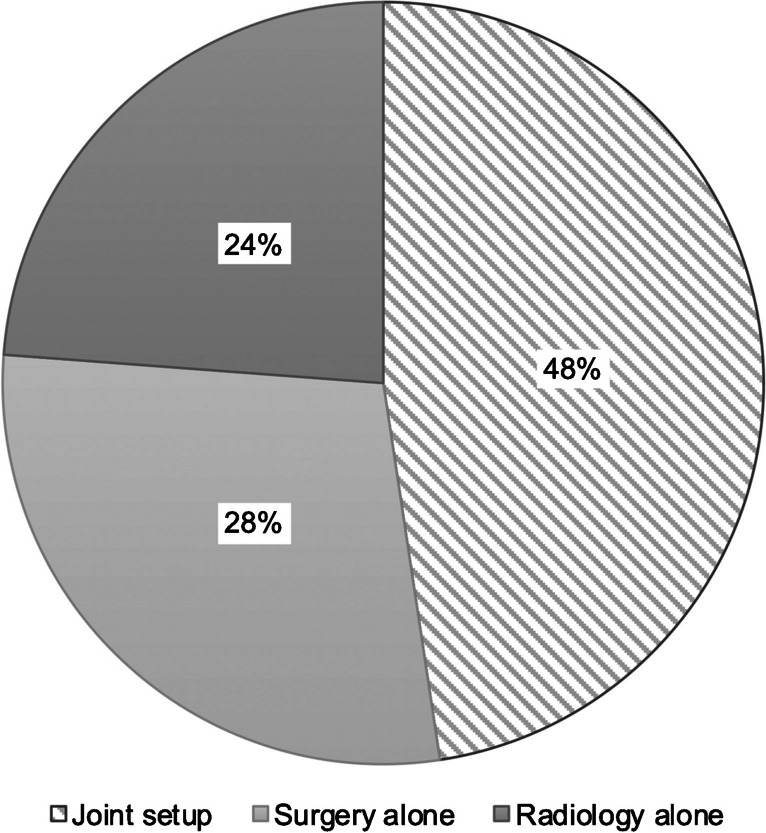
Usual setup for liver transplantation intraoperative ultrasound

Intraoperative US is performed at most sites as a joint examination (10/21 sites, 47.6%): The probe is handled by surgeons while the US machine is operated either by radiologists (8/10 sites, 80%), anesthesiologists (1/10 sites, 10%), or gastroenterologists (1/10 sites, 10%). Two institutions (9.5%) begin by using a joint setup and then switch to an US examination performed by radiology alone after abdominal wall closure. At five of 21 sites (23.8%), the intraoperative US is solely in the hands of radiology, including intra-abdominal probe handling. At six of 21 sites (28.6%), intraoperative US is performed by surgeons alone; radiology may be consulted if needed (two sites, 9.5%) (Fig. 1).

### Timing of intraoperative controls

Eighteen of 21 sites (85.7%) use intraoperative US in every patient with pre-scheduled time points during liver transplantation. Yet, the timing and the frequency at which the controls are applied varies between the centers. The number of scheduled US controls ranges between one to four examinations (median three examinations). The most used time points for scheduled intraoperative US controls are immediately before or after abdominal wall closure in 16/18 centers, 88.9%, and 18/18 centers, 100%, respectively. The third commonly used time point is immediately after arterial reperfusion and scheduled by 13/18 centers (72.2%). Three centers (16.7%) additionally use US controls after the biliary anastomosis is completed to check if adequate perfusion status is maintained. At three of 21 sites (14.3%), intraoperative US is only performed when surgeons have doubts about vascular patency, without following specific timing or frequency schedules.

### Documentation

**Fig. 2:**
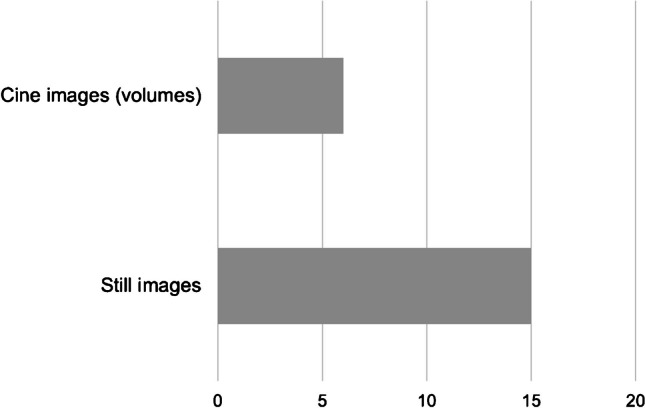
Type of images documented during intraoperative ultrasound

Most sites (13/21 sites, 61.9%) always store intraoperative US images for subsequent reassessment and reporting. The digital storage practice varies depending on the setup, with 84.6% (11/13 sites) performing storage when US is conducted in collaboration with radiology or by radiology alone, and 25% (2/8 sites) when performed solely by surgery or in collaboration with gastroenterology or anesthesiology. Two sites intermittently store intraoperative US images when radiology is consulted, which deviates from their initial setup. Representative still images of the liver transplant, including flow images and velocity measurements, are commonly documented following established standards. Additionally, six out of 21 sites (28.6%) routinely engage in more extensive documentation by storing 2-dimensional (D) or 3-D US volumes (cine sweeps) (Fig. 2).

## Discussion

This paper presents results of a multicenter survey among the European sites for pediatric liver transplantation, investigating current practices regarding intraoperative imaging. The pre- and postoperative findings are reported separately [19, 20]. This survey shows that intraoperative US is a commonly applied method to assess the quality of the vascular anastomosis in order to ensure optimal perfusion of the liver transplant. Its regular use is supported by the literature proving that US monitoring can detect inadequate graft inflow and outflow early and thus assist surgical improvement [12–18, 21–25]. Yet, this survey also shows that intraoperative US monitoring is handled differently at the centers regarding the general setup, disciplines involved, applied US technique, image documentation, and monitoring frequency.

Most sites performing pediatric liver transplantation adopt a collaborative approach for intraoperative US. Within this joint setup, surgery handles the probe manipulation on the transplanted liver under sterile conditions, while radiology predominantly operates the US machine settings. Less commonly, the intraoperative US is done by surgery alone (28.6%) or by radiology alone (23.8%). One advantage of the joint setup with shared responsibilities is that imaging parameters can be more specifically adapted to the intraoperative situation which is known to be technically demanding [25–27]. Flow disturbances at the anastomotic sites, increased portal venous velocity similar to arterial velocities, hepatic buffer response, high heart rates, and small vessel size in young children can complicate vascular detectability and need to be addressed by adapting scan parameters or equipment [18, 28]. However, in a two-operator joint setup, skillful probe handling coordinated with machine operation needs precise communication and training. A future recommendation on the favored setup needs to take personal resources and expertise at the site as well as technical prerequisites into account.

Among the enters, color Doppler US served as the basic, most frequently used vascular imaging technique followed by power angio mode, and B-flow. Non-Doppler-based techniques like B-flow are less angle-dependent and have higher temporal as well as spatial resolution with potential advantages to delineate small vessels during the transplantation [29, 30]. Despite its problem-solving capacity for assessing challenging vascular supply, the intraoperative use of CE-US is currently only implemented at three European centers [31].

It is noteworthy that only 61.9% of the European sites always perform digital storage of the intraoperative images. The documentation rate is substantially higher at sites where radiology is part of the intraoperative US monitoring setup providing access to picture archiving and communication systems. Storage of the intraoperative images and their general availability for later comparison during the postoperative window is important as they can serve as a roadmap, providing anatomical detail regarding transplant type and vascular connections in addition to the surgical report. The quality of intraoperative documentation can be further improved by storing US volumes in addition to representative single still images, thus achieving a more complete overview of the often-tortuous vascular course. However, this is currently employed by only a limited number of institutions (28.6%).

Quantitative Doppler US values are used to identify potential problems with graft in- and outflow during the operation. However, the way quantitative US scores are incorporated into clinical decision-making is still largely dependent on local expertise. Only a few systematic reviews including smaller case series have been published lacking general applicability of normal values and defined cutoff scores at defined time points [18, 32]. The intraoperative values can be utilized as a baseline for specific, individual follow-up and carry prognostic information. For example, an increased risk for early hepatic artery thrombosis has been associated with the presence of lower intraoperative peak systolic velocities (< 40 cm/s) and resistive indices (< 0.6) [17]. Likewise, increased acceleration times and a *tardus parvus* flow pattern were linked to the presence of hepatic artery stenosis in children and adults arguing for closer postoperative monitoring interval at follow-up [33].

This survey also highlights considerable differences regarding the timing and frequency of the intraoperative US. At the European institutions, the number of scheduled intraoperative controls varies between sporadic use and four examinations (median three examinations). The most used time point to check the perfusion status is at the end of surgery after abdominal wall closure. Yet, most sites also perform prior controls starting with arterial reperfusion. Clearly, the benefit of performing multiple controls during the operation and starting as early as after reperfusion is that potentially deleterious abnormalities like hepatic artery thrombosis are found early and can be swiftly revised [22]. Also, comparison with initial values allows a better estimation of the pressure changes on hepatic perfusion provoked by abdominal wall closure [25]. A potential downside of using multiple intraoperative controls may be the risk of overinterpretation of early flow disturbances attributable to transitional changes like arterial spasm which may self-resolve during the operation. Moreover, performing multiple US controls during the transplantation is very time-consuming, especially with a joint or radiology alone setup, and requires trained staff who have to be released from other duties.

In conclusion, the survey shows that intraoperative US is consistently utilized across all sites during pediatric liver transplantation, but also identifies significant variations in terms of the US setup, technique preferences, timing of controls, and documentation practices. These differences provide valuable insights for future optimization and harmonization studies to find the best possible setup for intraoperative US during pediatric liver transplantation and how it should interlink with preoperative evaluation [19] as well as postoperative monitoring [20]. Moreover, the evaluation of alternative non-Doppler-based US techniques and CE-US for specific scenarios should be considered.

### Supplementary information

Below is the link to the supplementary material.

Supplementary file1 (PDF 145 KB)

## Data Availability

The datasets generated during and analyzed during the current survey research are not publicly available as individual privacy was guaranteed to all participating centers. Blinded data are however available from the authors upon reasonable request and with permission of all participating centers.
