# Supplementary material for: European Society of Pediatric Radiology survey of perioperative imaging in pediatric liver transplantation: (2) intraoperative imaging
Source: Pediatr Radiol. 2024 Jan 13;54(2):269–75. doi: 10.1007/s00247-023-05840-1 (PMC10830587; doi:10.1007/s00247-023-05840-1)
Supplement: Supplementary file 1 — Supplementary file1 (PDF 145 KB) [file 247_2023_5840_MOESM1_ESM.pdf]

ESPR survey on peri-operative imaging in children with liver transplantation

Aim

The European Society for Paediatric Radiology (ESPR) Abdominal Work Force Group in collaboration with European centres engaged in the field of paediatric liver transplantation aims to publish a position paper regarding diagnostic imaging recommendations. The collaboration intends to identify a group of key sites to harmonize and improve imaging pre- and post-transplantation in children and may later want to cooperate in data acquisition and multicentre studies.

The survey

This survey focuses on the current status regarding imaging procedures including elastography in paediatric liver transplantation. For each site, a person responsible for the local transplantation program will be primarily addressed and asked to fill out the survey as a representative of their centre. As some of the questions are quite specific please involve the sub-specialists of your team whenever necessary.

In this online survey you can use the backwards and forwards button to switch between each section. The filled-in answers will be remembered and can be altered unless you close or finalize the document. Also, a printable PDF version of the survey is attached to your invitation email. The PDF version can be used in preparation and easily handed round your team. If you prefer, you can also send the filled-out paper version of the survey per fax, email or mail to my address below.

All information provided by you will be treated confidential. In case of a publication, approval will be obtained by all participating centres and co-authorship will be offered.

Please do not hesitate to contact me if you have any questions.

[redacted]

[redacted]

My institution

Please fill in your name and full contact details.

Name
First and last name

Meine Antwort

Medical specialty

- ☐ Paediatric transplantation surgery
- ☐ Paediatric gastroenterology
- ☐ Paediatric intensive care
- ☐ Paediatric radiology/radiology

Name of your institution

Meine Antwort

Email

Meine Antwort

Phone number

Meine Antwort

Which setting best describes your workplace?

- ○ University / Academic hospital setting
- ○ Specialist childrens' hospital setting
- ○ Community / District hospital setting
- ○ Sonstiges: ______

Please fill in the responsible persons for each subspecialty at your site (name and email address)

Paediatric transplantation surgery
Name and email address of the responsible person(s)

Meine Antwort

Paediatric gastroenterology
Name and email address of the responsible person(s)

Meine Antwort

Paediatric intensive care
Name and email address of the responsible person(s)

Meine Antwort

Paediatric radiology/radiology
Name and email address of the responsible person(s)

Meine Antwort

Demographics

1. How many children < **18 years** of age were liver transplanted on average per annum in the last three years (2018-2020)?
Please give a number.

Meine Antwort

2. How many children < **6 years** of age were liver transplanted on average per annum in the last three years (2018-2020)?
Please give a number.

Meine Antwort

3. How many children < **1 year** of age were liver transplanted on average per annum in the last three years (2018-2020)?
Please give a number.

Meine Antwort

4. What proportion of children < **18 years** of age were liver transplanted for treatment of metabolic disease on average per annum in the last three years (2018-2020)?
Transplanted for metabolic disease in %

Meine Antwort

5. What was the proportion of children < **6 years** with whole liver transplantation on average per annum in the last three years (2018-2020)?
Whole liver transplants in %

Meine Antwort

6. What was the proportion of children < **6 years** of age with living-related liver transplantation on average in the last three years (2018-2020)?
Living related transplantation in %

Meine Antwort

7. What was the proportion of children < **6 years** with secondary abdominal closure by PATCH interposition on average in the last three years (2018-2020)?
Patch interposition in %

Meine Antwort

Pre-transplant imaging

8. Do you (as a representative of your center) agree that ultrasound is the preferred imaging modality for definition of anatomy and to plan liver transplant surgery in children?

- ○ No
- ○ Yes

9. Do you perform abdominal CT imaging in children in preparation of liver transplantation?

- ○ No
- ○ Yes, in all patients
- ○ Yes, in selected patients - please specify below

- ☐ In children < 1 year
- ☐ In children < 6 years
- ☐ In children not requiring sedation
- ☐ In children with prior abdominal surgery (e.g. Kasai)
- ☐ In children with abnormalities on US
- ☐ Sonstiges: ______

If you answered "yes" to the previous question please continue with question #10. If you answered "no" please jump to question #13.

10. Please check your main indications for CT in pre-transplant evaluation

- ☐ Vascular anatomy
- ☐ Vascular patency
- ☐ Organ sizes
- ☐ Tissue and lesion characterization
- ☐ Sonstiges: ______

11. Please specify the usual CT scan area

- ○ Upper abdomen (liver)
- ○ Complete abdomen

12. Please **mark all** the CT phases you perform

- ☐ Native (no contrast)
- ☐ Arterial phase
- ☐ Parenchymal phase
- ☐ Late phase

13. Do you perform abdominal MRI imaging in children in preparation of liver transplantation?

- ○ No
- ○ Yes, in all patients
- ○ Yes, in selected patients - please specify below

- ☐ In children < 1 year
- ☐ In children < 6 years
- ☐ In children not requiring sedation
- ☐ In children with prior abdominal surgery (e.g. Kasai)
- ☐ In children with abnormalities on US
- ☐ Sonstiges: ______

If you answered "yes" to the previous question please continue with question #14. If you answered "no" please jump to question #15 on the next page.

14. Please check your main indications for MRI in pre-transplant evaluation:

- ☐ Anatomy
- ☐ Vascular patency and flow
- ☐ Organ sizes
- ☐ Tissue characterization (e.g. iron overload, fat quantification)
- ☐ Lesion characterization
- ☐ Sonstiges: ______

15. Which MR vascular imaging technic do you use:

	2D	3D	Time-resolved / 4D (temporal resolution below 5 seconds)
Contrast-enhanced MR-Angiography (CE-MRA)	☐	☐	☐

	TOF	bSSFP	ASL	PCA	4D Flow	Other
Non-contrast MR-Angiography (NCE-MRA)	☐	☐	☐	☐	☐	☐

TOF – time of flight; bSSFP – balanced steady state free precession (True FISP, Fiesta, b-FFE, B-Trance); ASL – arterial spin labeling; PCA – phase contrast angiography)

Intra-operative imaging

16. Do you use intra-operative ultrasound to control your vascular anastomoses?

- ○ No
- ○ Yes, in every patient
- ○ Yes, in some patients - please specify below

Meine Antwort

If you answered "yes" to the previous question please continue with question #17. If you answered "no" please jump to question #24 on the next page.

17. Who performs the intra-operative ultrasound?

- ☐ Transplant surgeon alone
- ☐ With gastroenterologist
- ☐ With radiologist
- ☐ With anaesthesiologist

18. Do you safe and store intraoperative ultrasound images to document the results?

- ○ No
- ○ Yes, in every patient
- ○ Yes, when performed by radiologist
- ○ Sonstiges: ______

19. Do you safe and store cine images (2D/3D volumes) to document the intra-operative results?

- ○ No
- ○ Yes, in every patient
- ○ Yes, when performed by radiologist
- ○ Sonstiges: ______

20. Do you perform intra-operative US at defined time points during the operation?

- ☐ No
- ☐ Yes, after hepatic artery anastomosis
- ☐ Yes, after biliary anastomosis
- ☐ Yes, before and after abdominal closure
- ☐ Sonstiges: ______

21. Which US based vascular imaging technique do you use intra-operatively?

- ☐ Color Doppler
- ☐ Power angio mode
- ☐ B-Flow
- ☐ Microvascular flow
- ☐ Sonstiges: ______

22. Do you additionally use US contrast intra-operatively?

- ○ No
- ○ Yes, in every patient
- ○ Only in case of unclear situations

23. Do you use other modalities or devices to monitor your vascular anastomoses?

- ○ No
- ○ Yes
- ○ Sonstiges: ______

Post-transplant imaging

24. Do you perform post-operative ultrasound monitoring on the ICU within the first 7 days?

- ○ No
- ○ Yes, in every patient
- ○ Yes, in some patients - please specify below

Meine Antwort

25. Is the intra-operative information about the liver transplantation (graft type, vascular and biliary anastomosis, intra-operative problems) provided to you before the 1st US examination?

- ☐ No
- ☐ Yes, in form of the surgical report
- ☐ Yes, as a surgical drawing on paper
- ☐ Yes, by stored images of the intra-operative US examination
- ☐ Sonstiges: ______

26. When is the 1st post-operative ultrasound on the ICU scheduled?

- ○ After arrival on ICU
- ○ Within 6-12 hours
- ○ Within 24 hours
- ○ Next working day (max. 72 hours)
- ○ In case of intra-operative or clinical abnormalities
- ○ No general rule

27. Is there a defined frequency for ultrasound monitoring in the early post-operative phase (within the first 7 post-operative days)?

- ○ No
- ○ Twice daily (approx. every 12 hours)
- ○ Once daily (every 24 hours)
- ○ Every 48-72 hours
- ○ Sonstiges: ______

28. Do you regularly perform ultrasound follow-up examinations after the acute post-operative phase ended (>7 days after the transplantation)?

- ○ No, only in case of abnormalities
- ○ Yes, on a daily basis until discharge
- ○ 2-3 times a week
- ○ Every week
- ○ Sonstiges: ______

29. Who mainly performs the post-operative ultrasound monitoring?

- ☐ Transplant surgeon
- ☐ Gastroenterologist
- ☐ Intensive care doctor
- ☐ Radiologist
- ☐ Sonographer
- ☐ Interdisciplinary team

Please specify in case more than one answer or interdisciplinary teams are marked.

Meine Antwort

30. Indicate the level of experience of the person primarily performing the post-operative ultrasound monitoring

- ○ 1st - 2nd year resident
- ○ 3rd - 5th year resident
- ○ Fellow
- ○ Consultant

31. Does your center use a standardized abdominal protocol for post-transplant ultrasound?

- ○ No
- ○ Yes

If you answered "yes" to the previous question please continue with question #32. If you answered "no" please jump to question #34.

32. Please mark the key imaging elements which are documented in your standardized protocol

	LIVER PARENCHYMA (B-MODE, GREY-SCALE)
Representative still images of neohilum and outflow (curved probe)	☐
Cine-sweeps covering area from outflow to neohilum (curved probe)	☐
Representative high-resolution still images (linear probe)	☐
Cine-sweeps high-resolution (linear probe)	☐
Liver length measurement	☐
Liver elastography	☐

	LIVER VASCULAR (FLOW IMAGES, E.G. COLOR DOPPLER)
Representative still images at neohilum and outflow (curved probe)	☐
Cine-sweeps covering area from outflow to neohilum (curved probe)	☐
Flow velocities/indices at porta hepatis and outflow (curved probe)	☐
Representative high-resolution still images (linear probe)	☐
Cine-sweeps high-resolution (linear probe)	☐
Flow velocities/indices at intrahepatic level (linear probe)	☐

	EXTRAHEPATIC LOCATIONS
Spleen length measurement	☐
Spleen flow velocity/indices at hilum	☐
Spleen elastography	☐
Pleural effusion	☐

Ascites ☐

OTHER ELEMENTS?
Please specify

Meine Antwort

33. Which ultrasound flow imaging technique do you use?

- ☐ Color Doppler
- ☐ Power Angio
- ☐ B-Flow
- ☐ Microvascular flow
- ☐ Sonstiges: ____

34. Do you routinely use US contrast examinations for post-transplant evaluation?

- ○ No
- ○ Yes - please tell us below for what indications

Meine Antwort

35. Do you use other non-imaging based devices to control vascular anastomoses and vascular patency?

- ○ No
- ○ Yes - please specify below

Meine Antwort

36. Do you perform abdominal CT postoperatively after LTX?

- ○ No
- ○ Yes - please mark below for which indications

- ☐ Hepatic artery thrombosis, suspected
- ☐ Hepatic artery stenosis, grading
- ☐ Portal vein thrombosis, suspected
- ☐ Portal vein stenosis, grading
- ☐ Hepatic outflow obstruction
- ☐ Unclear intraabdominal fluid collections (seroma, bilioma, abscess), +/- in combination with CT guided drainage
- ☐ Sonstiges: ____

37. How often did you perform abdominal CT in children after liver transplantation in the last year?
Approximate number

Meine Antwort

38. Do you perform MRI after liver transplantation and before discharge from the hospital?

- ○ No
- ○ Yes - please mark below your indications

- ☐ Bile duct abnormalities on US
- ☐ Characterization of intra-abdominal fluid collections on US
- ☐ Suspected vascular problems on US
- ☐ Post-operative baseline examination before discharge in children not requiring sedation

Liver elastography

PLEASE FILL IN THIS SECTION ONLY IF YOUR CENTER PERFORMS IT.

39. Which ultrasound elastography technique do you use?

- ☐ Transient elastography
- ☐ Point shear wave elastography (pSWE)
- ☐ Two-dimensional shear wave elastography (2D-SWE)

40. When is the 1st post-operative ultrasound liver elastography scheduled?

- ○ No general rule
- ○ Within 24 hours
- ○ Next working day (max. 72 hours)
- ○ Within the first 7 days
- ○ Within the hospital stay/before discharge

41. Is there a defined frequency for ultrasound liver elastography in the early post-operative phase (within the first 7 post-operative days or until discharge from the ICU)?

- ○ No general rule
- ○ Twice daily (approx. every 12 hours)
- ○ Once daily (every 24 hours)
- ○ Every 48-72 hours
- ○ Sonstiges: ____

42. Do you require children to have fasted before the ultrasound examination?

- ○ No
- ○ Yes, all children minimum 2 hours
- ○ Yes, all children minimum 4 hours
- ○ Sonstiges: ____

43. Do you control the amount of transducer pressure applied in freehand SWE examinations with epigastric probe position (directly above the liver)?

- ○ No, probe pressure is not controlled
- ○ No, probe pressure is not controlled, but lowest possible transducer pressure must be used
- ○ Yes, semi quantitatively by measuring the degree of abdominal compression (Distance from skin to lower transplant border or spine)
- ○ Yes, by measuring compressive transducer force (technical device)

44. Do you perform MR Elastography in children after LTX?

- ○ No
- ○ Yes

Outlook

45. Would you be interested to standardize your peri-operative imaging approach together with other European centers?

- ○ No
- ○ Yes

46. Would you be willing to share your ultrasound protocols presently applied?

- ○ No
- ○ Yes

47. Would you be interested to work with us on a common standard how to perform peri-operative ultrasound in children with liver transplantation?

- ○ No
- ○ Yes

48. Would you be interested to collect standardized imaging data of liver transplanted children within a European network?

- ○ No
- ○ Yes

Link abrufen

Geben Sie niemals Passwörter über Google Formulare weiter.

Dieser Inhalt wurde nicht von Google erstellt und wird von Google auch nicht unterstützt. Missbrauch melden - Nutzungsbedingungen - Datenschutzerklärung

Füllen Sie die Antwortfelder vorab aus und klicken Sie dann auf "Link abrufen"

Google Formulare
